# Quality of Life and Mental Health Well-Being in Sjögren’s Disease in the UK: A Cross-Sectional Comparative Analysis

**DOI:** 10.3390/jcm14061939

**Published:** 2025-03-13

**Authors:** Minan Y. Al-Ezzi, Khalid S. Khan, Anwar R. Tappuni

**Affiliations:** 1 Bristol Dental School, Faculty of Health and Life Sciences, University of Bristol, Bristol BS2 0PT, UK; m.al-ezzi@bristol.ac.uk; 2 College of Medicine and Dentistry, Ulster University, Birmingham B4 6BN, UK; 3 Department of Preventive Medicine and Public Health, Faculty of Medicine, University of Granada, 18016 Granada, Spain; profkkhan@gmail.com; 4 Faculty of Medicine and Dentistry, Queen Mary University of London, London E1 1BB, UK

**Keywords:** quality of life, oral health-related quality of life, anxiety, depression, Sjögren’s disease, well-being

## Abstract

**Objectives:** We aimed to evaluate the quality of life (QoL), oral health-related quality of life (OHRQoL), and mental health well-being in female patients diagnosed with Sjögren’s Disease compared with healthy controls. **Methods:** An ethically approved cross-sectional study was carried out on 65 female patients with a confirmed diagnosis of Sjögren’s Disease according to the American European Association Consensus Group Criteria and 61 sex-matched healthy volunteers. The World Health Organization Quality of Life-BREF, Oral Health Impact Profile-14, and Hospital Anxiety and Depression Scale were used to evaluate the general and oral health-related QoL (OHRQoL) and the mental health well-being of the participants. **Results:** The general QoL was lower in the patients’ group (*p* < 0.05) compared with the control group in all four domains (physical, psychological, social, and environment). The OHRQoL was significantly reduced in the patients’ group, who were more anxious (58.5%, *n* = 38/65) and four times more depressed (32.3%, *n* = 21/65) compared with healthy volunteers (anxiety = 21%, *n* = 13/61; depression = 8.2%, *n* = 5/61). **Conclusions:** This study concludes that Sjögren’s Disease negatively affects QoL and mental well-being. Therefore, addressing these aspects in patients’ management is crucial to helping individuals cope with the disease’s burden and ultimately enhancing their overall life experience.

## 1. Introduction

Sjögren’s Disease is an inflammatory autoimmune rheumatic disorder that affects the exocrine glands, especially salivary and lacrimal glands, causing persistent oral and lacrimal dryness, which are the main characteristic features of SD [1]. Sjögren’s Disease mostly affects females with a ratio of 9:1, where the majority of cases are diagnosed in the fifth or sixth decade of life [2,3]. The treatment is essentially palliative for this condition, and the clinical management entails symptomatic treatment to help improve physical symptoms that can enhance the quality of life (QoL) and mental health well-being of patients [4].

Studies have shown that patients affected by Sjögren’s Disease experience a substantial burden of the disease due to sicca symptoms (dry eyes and mouth in the absence of autoimmune disease), fatigue, and pain. These challenges can significantly impact patients’ ability to cope with daily activities, leading to a decline in both quality of life (QoL) and mental health well-being [5,6,7]. Symptoms such as persistent dry eyes and mouth, sexual dysfunction, joint pain, and fatigue are known clinical manifestations of the disease that can negatively interfere with the overall QoL of patients [5,8,9]. There is an adverse association between comorbidities, including anxiety and depression, with Sjögren’s Disease that can affect the mental health well-being in this population [10,11]. Recent large studies have revealed that anxiety and depression are symptoms in patients with Sjögren’s Disease that can affect QoL; however, the lack of control groups for comparison and the inclusion of patients regardless of whether or not they are diagnosed with another autoimmune comorbidity make it difficult to establish the internal validity of these studies and to confirm causation and correlation [12,13].

Saliva plays a pivotal role in preserving and maintaining oral health; therefore, when the amount or quality of saliva declines over time, a wide range of oral health problems can arise [14]. Dental caries, oral ulcers, fungal infections, swallowing, and speaking difficulties along with chemosensory problems, including smell and taste dysfunction, are some of the most common oral symptoms associated with Sjögren’s Disease [15,16,17]. Therefore, patients with Sjögren’s Disease can have compromised oral health that can influence OHRQoL. Several single-centre studies evaluated the oral health-related QoL (OHRQoL) and oral health status in patients diagnosed with Sjögren’s Disease compared to the controls [18,19,20]. The findings of these investigations were consistent in terms of the affected OHRQoL and deteriorated oral health status. However, the small sample size recruited, and the single-centre recruitment of patients with Sjögren’s Disease, limited the generalisability of the results. There is a need for larger studies to support healthcare practitioners managing patients with Sjögren’s Disease and provide coping strategies to improve their well-being and quality of life. The literature lacks evidence evaluating the association between Sjögren’s Disease and QoL and mental health well-being in a well-defined cohort of patients in comparison with healthy controls. Understanding patients’ overall QoL and mental health well-being will help healthcare practitioners to encompass patients’ needs in order for them to live a better life. We hypothesise that patients with a diagnosis of Sjögren’s Disease display impaired QoL and mental health well-being. Therefore, the aim of the current study is to evaluate the overall QoL, OHRQoL, and mental health well-being in patients diagnosed with Sjögren’s Disease.

## 2. Methods

The study outline and assessment methods used were reviewed by a patient representative at Barts Health in East London, and this study was approved by the London Bridge Research Ethical Committee (Reference number: 15/LO/2064, 10 February 2016). This study was conducted in the Multidisciplinary Sjӧgren’s Syndrome Clinic, Institute of Dentistry (IoD), Queen Mary University of London. The study adopted Wilson and Cleary’s (1995) conceptual model of patients’ outcomes that was revised and simplified later by Ferrans et al. (2005) and structured according to the STROBE statement checklist for combined studies (case–control and cross-sectional studies) [21,22,23]. One investigator conducted all assessments in no particular order for all participants. Updated medical and social history was obtained from all participants regarding their general and oral health, age, medications, fatigue, mouthwash, oral appliances, smoking, alcohol consumption, and disease duration. The overall QoL of the previous two weeks was evaluated for all participants by 26 items using the validated World Health Organisation Quality of Life-BREF (WHOQoL-BREF) [24]. The first question assessed the self-perceived QoL, “How would you rate your quality of life?”, whilst the second question assessed satisfaction with health, “How satisfied are you with your health?”. The first two questions were given a maximum score of five, to indicate the best QoL. The remaining 24 items assessed individuals’ QoL in four domains: physical health, psychological, social relationships, and environment. The items were rated on a Likert scale of one to five in each domain score. Raw domain scores were transformed to a 0–100 score according to the guidelines [24]. The higher the score, the better the QoL was perceived to be. A cut-off value of <60 indicating poor/unsatisfactory QoL was used [25].

The OHRQoL was assessed using Oral Health Impact Profile-14 (OHIP-14) within the previous twelve months’ period [26]. This assessing tool comprised fourteen items distributed into seven domains. The domains were functional limitation, physical pain, psychological discomfort, physical disability, psychological disability, social disability, and handicap. Responses were given on a five-scale rating: 0 = never, 1 = hardly ever, 2 = occasionally, 3 = fairly often, and 4 = very often. Severity was calculated by summing the scored 14 items (range 0–56) to obtain the total score of a participant, where a higher score denotes worse oral health QoL [27,28]. The mean value of items that comprised a domain was calculated to obtain each domain score [29]. The prevalence of oral health problems was estimated by calculating the percentage of respondents rating one oral health problem or more with “Fairly often” or “Very often” [30]. The extent of oral health problems was calculated by counting the number of items rated with “Fairly often” or “Very often”. The severity was calculated by summing up the scored 14 items (range 0–56) to obtain the total score of a participant, where a higher score denotes worse oral health QoL [27,28].

Mental health well-being was measured using the Hospital Anxiety and Depression Scale (HADS) [31]. This assessing tool comprised two domains, anxiety (HAD S-A) and depression (HADS-D). Each domain consists of seven items, with four coded responses that range from zero to three. A simple final sum for each domain was given to the final domain scoring value, where higher scores denote severity levels. A score ranging from 0 to 7 indicates a normal case, 8–10 = mild, 11–15 = moderate, and ≥16 = severe [32]. All the necessary permissions and licence required for using the questionnaires were obtained.

All questionnaires were checked for uncoded items and, if applicable, participants were requested to complete the form. The assessment was considered invalid when responses of more than 20% of data were missing.

### 2.1. Study Group

The Multidisciplinary Sjӧgren’s Clinic hosted this study at the Institute of Dentistry (IoD), Queen Mary University of London, UK. Patients were managed and treated by a multidisciplinary team composed of a Rheumatologist, Oral Physician, and Ophthalmologist. Female patients diagnosed with Sjӧgren’s Disease according to the American European Consensus Group (AECG) criteria were deemed eligible to take part in the study [33]. Patients were recruited during the period between 2 March and 30 November 2016 from the above clinic or identified after screening its research clinical database of 337 rheumatic patients. A postal invitation pack with detailed information on the research project was sent to the eligible patients. The project was also advertised on the British Sjögren’s Syndrome Association (BSSA) website, and members with Sjögren’s Disease diagnoses who were interested to take part were able to contact the research team.

This project was advertised in the IoD, and eligible sex-matched healthy controls who were at least 18 years old, capable of consent, and understanding verbal explanations in English consented to take part. Excluded were subjects with a current cold or blocked nose and those who had head or neck radiation, chemotherapy, a diagnosis of salivary gland disease or swelling, Sjögren’s Disease associated with other autoimmune rheumatic diseases (previously known as secondary Sjögren’s Syndrome), including lupus erythematosus, uncontrolled diabetes, asthma, lichen planus, allergic sinusitis, pregnancy, breast feeding, significant dental problems, and candidiasis. A record of the medications taken by eligible participants was kept to assess whether there was a correlation with QoL.

### 2.2. Statistical Analyses

Data were analysed using the Statistical Package for Social Sciences, IBM Corporation, SPSS Inc., Chicago, IL, USA, version 23 statistical software. A pilot study based on a mean difference in the smell and taste outcome of a larger study was conducted to help estimate the calculation of the sample size. The nomogram method was used to estimate the sample size at the power calculation based on a mean difference that was set at 90%, and the distribution of the data was determined via the Shapiro–Wilk test, and the level of significance was set at 5%. A total of 75 subjects (cases and healthy volunteers) were required to detect a level of mean difference. The sample was inflated by 20% to give a total of 90 participants (45 cases and 45 healthy volunteers) to accommodate for dropout. Continuous variables were expressed as mean followed by ±SD, and the mean difference was followed by a 95% confidence interval (CI). Independent t-test and Chi-square tests were used. Multivariate linear regression analyses were used in the patients’ group to control confounders including fatigue, smoking, alcohol consumption, mouthwash users, oral appliances, and medications (categorical variables), age, and disease duration (continuous variables). Frequency analysis was used to determine the rate of the self-reported symptoms affecting patients’ QoL. All *p*-values were reported for transparency regardless of significance.

### 2.3. Patient and Public Involvement

The project design and protocol, including the questionnaires, were reviewed by a patient representative who was a member of the Patient and Public Engagement Group at the Faculty of Medicine and Dentistry, QMUL, before the research project started.

## 3. Results

A total of 65 patients (mean age = 59 ± 13) and 61 healthy participants (mean age = 43 ± 15) were eligible and consented to take part in this study. The advantage of recruiting more participants than that obtained from the power calculation was the high power of the multivariate regression analysis, to avoid spurious or false statistical significance due to over drafting. Table 1 illustrates the participants’ demographics; they were all literate with different levels of educational attainment. The ethnicity of the patients included 69.23% from a White background, 10.76% Black, 13.84% Asian, 1.53% mixed or multiple backgrounds, and 4.61% from other ethnic groups. The ethnicity of the healthy volunteers included 46.77% from a White background, 8.06% Black, 20.96% Asian, 19.35% mixed or multiple backgrounds, and 4.83% from other ethnic groups. Retired patients and controls made up 53.84% and 9.67% of the population, respectively. Employed patients (30.76%) and healthy volunteers (69.35%) were higher than unemployed patients (15.38% and 19.35%, respectively). No significant baseline differences were found in the characteristics between the patients and control groups, except in terms of employment status (*p*-value = 0.001). Disease duration ranged between 6 months and 17 years based on the patients’ reporting of their early symptoms of the disease.

### 3.1. Impact on the General Quality of Life

The self-perceived assessment by WHOQoL-BREF of the overall QoL measured by the first global question “How do you rate your quality of life?” of the questionnaire was statistically significantly lower in the patients’ group (3.5, ±0.9) compared with that of the healthy volunteers’ group (4.3, ±0.5), with a mean difference of 0.8 and 95% CI = 0.6–1.1, indicating low QoL. Similarly, the second global question that assesses individual’s satisfaction of health “How satisfied are you with your health?” was statistically significantly lower in the patients’ group (2.8, ±0.9) compared with the healthy volunteers’ group (4.1, ±0.7) with a mean difference of 1.2 and 95% CI = 0.95–1.5. The results of the mean difference ±SD of the four domains of QoL assessed by WHOQoL-BREF indicated that the patients’ group had lower QoL compared to the healthy volunteers’ group (Table 2). None of the confounding factors contributed to the reduced QoL in the patients’ group.

1. Physical domain: This domain deteriorated in 54% of patients, compared to only 7% of the healthy participants who had their physical life quality affected negatively.
2. Psychological domain: The quality of this domain was low in 48% of patients, compared to only 10% of the healthy volunteers.
3. Social domain: The quality of this domain was low in 45% of the patients compared to only 21% of the healthy participants.
4. Environmental domain: This domain was compromised in 22% of the patients compared to only 10% of the healthy participants.

### 3.2. Impact on the Oral Health-Related Quality of Life

In the patients’ group, 69% (*n* = 45/65) demonstrated worse OHRQoL compared to 15% (*n* = 9/61) of the healthy volunteers’ group. The results also showed that all seven domains of OHRQoL were worse in the patients’ group (Table 3). The prevalence, extent, and severity of the oral health problems in this study’s total population are illustrated in Table 4. Out of all the examined confounding factors, only age (β = 0.4, *p* = 0.008) and alcohol (β = −0.3, *p* = 0.01) displayed a significant association with the total score of OHRQoL, whilst mouthwash contributed significantly to the ‘’functional limitation’’ domain only (Table 5).

### 3.3. Impact on Mental Health Well-Being

In the patients’ group, 58.5% (*n* = 38/65) appeared to be statistically significantly more anxious compared with the healthy volunteers’ group (21%, *n* = 13/61). Depression symptoms were statistically significantly worse in the patients’ group (32.3% *n* = 21/65) compared with the healthy volunteers’ group (8.2% *n* = 5/61). Disease duration did not contribute to anxiety or depression symptoms in the patients’ group. Table 2 illustrates the mean and mean difference ±SD between the patient and control groups.

## 4. Discussion

The purpose of this study was to assess the QoL and mental health well-being of patients diagnosed with Sjögren’s Disease. Our results demonstrated that patients with Sjögren’s Disease had deteriorated QoL and mental health well-being. The current study was part of a larger study that assessed the impact of Sjögren’s Disease on clinical symptoms including smell, taste, and sexual function; therefore, participants had to attend physically to undergo clinical assessments and respond to surveys. Disease duration was difficult to establish as the majority of patients had symptoms for years before they presented to the clinic. The age and sex distribution of the study group reflects the typical Sjögren’s Disease population, which is more than 90% female and predominantly postmenopausal.

In the present study, the QoL of the patients with Sjögren’s Disease was significantly impacted in all domains—the physical, psychological, social, and even environment domains—compared with the healthy volunteers. The physical domain was more impacted in the patients’ group than other domains, emphasising the patients’ bodily struggles. Our results are aligned with previous research evaluating the quality of life of Sjögren’s Disease patients, which discovered that all areas were impacted for the patients except for the “environment” domain [34,35]. Our previous findings showed that sexual dysfunction in the patients’ group had a negative impact on the social domain, indicating that the patients’ social life quality was reduced by sexual impairment. Therefore, investigating and managing this aspect in patients with Sjögren’s Disease can improve overall QoL [8]. In the patients’ group, the consumption of alcohol was found to be related to the environment domain. This correlation could indicate that patients’ discontent with their surroundings contributed to their alcohol consumption.

Oral health-related quality of life is considered a relevant end-point criterion in evaluating the effects of a disease on individuals’ oral health over time. The oral health-related QoL was highly reduced in patients with Sjögren’s Disease compared with the healthy volunteers. Our results were consistent with previous studies, which reported oral distress in patients with Sjögren’s Disease compared with the controls [36,37,38]. The minimal important difference (MID = 13.7) in the OHIP-14 score between the patients and healthy volunteers’ groups was higher than the five scale units that were estimated earlier [39]. This shows that patients with Sjögren’s Disease demonstrated major deterioration in their oral health quality of life.

The prevalence, extent, and severity of oral health problems were significantly higher in the patients’ group compared with the healthy volunteers. However, patients with increased oral health problems did not necessarily equate to oral dryness, especially given that 23% of patients had intraoral appliances including night guards and partial or complete dentures, which could have contributed to their oral health problems.

We found that the most affected domain of OHIP-14 was “Functional limitation” in the patients’ group compared to the healthy volunteers. In the regression analysis, mouthwash contributed significantly to the compromised functional aspect of patients’ oral health, which can be an indication of underlying oral health condition in the patients’ group and a reflection of their desire to use mouthwash to improve their oral health.

Age and alcohol intake were common variables associated with the oral distress and physical disability of the patients’ oral health. These findings contradicted others who found no correlation between ageing and oral health quality [40,41]. As for alcohol intake, we found that it has an association with oral health problems, which could indicate an inverse relationship between OHRQoL and alcohol intake; however, more research is needed in this regard. To our knowledge, there has been no previous demonstration that these correlations were reported in this group of patients.

Our research revealed that patients’ mental health was noticeably worse than that of healthy volunteers, supporting the theory that Sjögren’s Disease is a chronic, debilitating condition that has an impact on patients’ bodily and mental well-being [40,42].

In this study, patients were more anxious and four times more depressed than the healthy volunteers. Our results were in line with others who found that the mental health well-being was significantly reduced in patients with Sjögren’s Disease [11,35,41,43,44]. Our study showed that anxiety symptoms in the patients’ group were more reported but not significantly different from that of the healthy volunteers. Unlike anxiety, depression was significantly higher in our patients’ group compared with the healthy volunteers. These findings supported others who reported that depression symptoms were more pronounced than anxiety in the patients’ group when assessed by HADS [11,41,45]. Only one small study (*n* = 24) reported that anxiety symptoms were significantly higher than depression symptoms in patients with Sjögren’s Disease compared with controls [46]. Meanwhile, other studies reported that anxiety and depression symptoms were equally present in patients with Sjögren’s Disease [35,43].

Our study has several strengths: The project was carried out at the UK’s largest trust, the Barts Health Trust, a major health service provider with the largest database. Our understanding of the feasibility of employing the questionnaires to avoid data loss in the present study came from the pilot project. Furthermore, the study report was organised in accordance with the STROBE statement checklist for combined studies (case–control and cross-sectional studies) [23]. This study’s power of 90% and the sample size (65 patients and 61 healthy volunteers) were sufficient to draw conclusions. The clinical criteria for diagnosing Sjögren’s Disease were based on the recommended AECG criteria, which ensured identifying a pure group of patients. The range of the validated questionnaires used and data collection by one researcher ensured that the necessary information was collected from participants and that performance bias was avoided.

The Wilson and Cleary (1995) model is the most widely cited conceptual framework of the HRQoL that integrates both the biological and psychological aspects of health outcomes [21,47]. The model provides a theoretical approach to conceptualising HRQoL as a multidimensional construct and is useful to guide the development of new theories. Therefore, we decided to apply the Wilson and Cleary approach in this study. The lack of the use of other models is noted as a limitation of this study. Our subgroup analyses may be underpowered, which is a limitation of this study; therefore, cautious interpretation is required. Also, consistency stats to assess internal validity were not used, which is another limitation of this study. There was potentially selection bias for patients entering the study, as patients were more likely to have had problems with dryness if they were seen at the Dental Hospital, and/or if they expressed interest in this study. The difference in the mean age between the patients’ group (59 years) and control group (43 years) was one of this study’s main limitations and could have resulted in the findings being overestimated. A more detailed matching sample would have required selection according to several variables, including age. As a result, we examined the data of a subgroup of our study participants who were in the same age range as the control group. We observed that the findings remained the same for the entire analysis (the results are not displayed). Another limitation lies in the use of HADS, which was originally designed as a diagnostic screening tool for symptoms of anxiety and depression, not as a well-being measure. However, the tool was found to be the best possible one for the purpose of this study in terms of effectiveness, validity, reliability, sensitivity, specificity, simplicity, and ease of use [48,49]. The existing evidence on psychological, medical, and lifestyle interventions should be reviewed to develop a consensus on a strategy to be included in the British Society for Rheumatology’s management guidelines [4]. This approach would enhance patients’ overall well-being. Despite reporting the differences in QoL scores between groups in the current study, the clinical significance remains unclear, which adds to the current limitations. Future research should investigate the thresholds for significant changes in QoL scores and discuss their implications for patients.

Summary and conclusion: This work is a hypothesis-generating one; our findings require confirmation in future independent studies. We present important evidence of the compromised general QoL, OHRQoL, and mental health well-being of patients with Sjögren’s Disease. Early diagnosis and understanding the impact of Sjögren’s Disease on daily activities can help patients develop better coping strategies that contribute to improving their QoL and mental health well-being.

## Figures and Tables

**Table 1:** Characteristics of patients and healthy volunteers.

Characteristics	Patients *n* = 65	Volunteers *n* = 62	*p*-Value	Chi-Square	Total *n* = 127
**Age**	**Mean (95%)** **59 (55.8–62.1)**	**Mean (95%)** **43 (39.2–46.8)**			**Mean (95%)** **51 (48.4–54)**
**Ethnicity**	***N*** **(%)**	***N*** **(%)**	**0.1**	**5.5**	***N*** **(%)**
White UK	41 (63)	14 (22)			55 (43)
White others	4 (6)	14 (22)			18 (14)
White total	45 (69)	28 (45)			73 (57)
Mixed	1 (1)	4 (6)			5 (3)
Asian	9 (13)	13 (20)			22 (17)
Black	7 (10)	5 (8)			12 (9)
Other	3 (4)	12 (19)			15 (11)
**Partner**			0.9	0.00	
Yes	45 (69)	43 (71)			88 (70)
No	20 (31)	19 (31)			39 (31)
**Education**		--	0.07	5.2	--
Primary	1 (1)	2 (3)			3 (2)
Secondary	23 (35)	11 (17)			34 (26)
Tertiary	41 (63)	49 (79)			90 (70)
**Monthly income**			0.5	2.5	
GBP 500–1000	7 (10)	7 (11)			14 (11)
GBP 1000–2000	18 (27)	19 (30)			37 (29)
More than GBP 2000	33 (50)	26 (41)			59 (46)
Preferred not to say	5 (8)	10 (16)			15 (11)
Missing	1 (1)	0			1 (0.7)
**Employment**			0.01	28.9	
Employed	20 (30)	43 (69)			63 (49)
Unemployed	10 (15)	12 (19)			22 (17)
Retired	35 (53)	6 (9)			41 (32)
Missing	0	1 (1)			1 (0.7)

**Table 2:** Comparison of percentage rates of the amount of impairment of QoL in the patients’ group compared with the healthy volunteers.

Quality of Life Patients, *n* = 65 Healthy Volunteers, *n* = 61	Mean, ±SD	Mean Difference (95% CI)	*p*-Value
**Physical domain ^1^** Patients Healthy volunteers	55.4, ±19 80, ±12.7	25 (19–30)	0.01
**Psychological domain** Patients Healthy volunteers	61.8, ±16 73.7, ±11.7	12 (7–16.7)	0.00
**Social domain** Patients Healthy volunteers	61.6, ±20 73.6, ±17.2	12 (5.2–18.7)	0.02
**Environmental domain ** Patients Healthy volunteers	69.5, ±16 75.6, ±12.8	6 (0.9–11.2)	0.03
**Mental health well-being** Patients, *n* = 65 Healthy volunteers, *n* = 61	**Mean, ±SD**	**Mean Difference (95% CI)**	***p*** **-Value**
**Anxiety ^2^** Patients Healthy volunteers	8, ±4 5.2, ±3.3	2.8 (1.5–4)	0.1
**Depression ^2^** Patients Healthy volunteers	6, ±4 2.4, ±2.5	3.5 (2.3–4.6)	0.04

^1^ Overall QoL ≥ 60 in a scale of 0–100. ^2^ Normal HADS scores < 8.

**Table 3:** Comparison of the OHIP-14 domains between the patients’ and healthy volunteers’ groups.

OHIP-14 Domains * Patients, *n* = 65 Healthy Volunteers, *n* = 61	Mean, ±SD	Mean Difference (95% CI)	*p*-Value
**Functional limitation** Patients Healthy volunteers	1.5, ±1.1 0.1, ±0.4	1.4 (1.1–1.7)	0.02
**Physical pain** Patients Healthy volunteers	1.8, ±1 0.8, ±1.2	1 (0.7–1.5)	0.03
**Psychological discomfort** Patients Healthy volunteers	1.9, ±1.2 0.9, ±0.9	0.9 (0.6–1.4)	0.04
**Physical disability** Patients Healthy volunteers	1.4, ±1 0.4, ±0.6	0.9 (0.7–1.2)	0.00
**Psychological disability** Patients Healthy volunteers	1.5, ±1 0.6, ±0.7	0.8 (0.6–1.2)	0.01
**Social disability** Patients Healthy volunteers	1, ±0.9 0.4, ±0.6	0.6 (0.4–0.9)	0.01
**Handicap** Patients Healthy volunteers	1, ±1 0.2, ±0.5	0.8 (0.5–1)	0.02
**Total score** Patients Healthy volunteers	20.4 ±11 6.7, ±6.6	13.7 (10.5–16.9)	0.03

* No oral health problems = never, hardly ever and occasionally vs fairly often and very often.

**Table 4:** The prevalence, extent, and severity of self-perceived oral health problems in the patients’ and healthy volunteers’ groups.

Variable	Patients’ Group *N* = 65	Healthy Volunteers’ Group *N* = 61	95% CI	*p*-Value
Prevalence (%)	69.2%	14%	0.4–0.7	0.03
Extent (mean score)	2.6	0.3	1.6–3.2	0.04
Severity (mean score)	20.4	6.7	10.5–16.9	0.00

**Table 5:** Impact of age and alcohol on OHRQoL measured by OHIP-14.

OHRQoL	Standardised Coefficients (Beta)		
	Age *p*-Value 95% CI	Alcohol *p*-Value 95% CI	Mouthwash *p*-Value 95% CI
Total OHIP	β = 0.4 *p* = 0.008 95% CI = 0.1–0.7	β = −0.3 *p* = 0.01 95% CI = −13–−1.4	β = 0.2 *p* = 0.07 95% CI = −0.6–11
Functional limitation	β = 0.3 *p* = 0.03 95% CI = 0–0.1	β = −0.2 *p* = 1 95% CI = −1–0.1	β = 0.3 *p* = 0.04 95% CI = 0.02–1.2
Physical pain	β = 0.3 *p* = 0.09 95% CI = 0–0.05	β = −0.15 *p* = 0.3 95% CI = −0.8–0.2	β = 0.2 *p* = 0.1 95% CI = −0.09–1
Psychological discomfort	β = 0.4 *p* = 0.03 95% CI = 0–0.06	β = 0.3 *p* = 0.04 95% CI = −1.2–−0.02	β = 0.1 *p* = 0.3 95% CI = −0.3–0.9
Physical disability	β = 0.5 *p* = 0.007 95% CI = 0–0.06	β = −0.3 *p* = 0.03 95% CI = −1–−0.05	β = 0.2 *p* = 0.1 95% CI = −0.1–0.9
Psychological disability	β = 0.3 *p* = 0.06 95% CI = 0–0.05	β = −0.3 *p* = 0.02 95% CI = −1.2–−0.1	β = 0.1 *p* = 0.4 95% CI = −0.2–0.8
Social disability	β = 0.3 *p* = 0.07 95% CI = 0–0.04	β = −0.3 *p* = 0.03 95% CI = −1–−0.1	β = 0.2 *p* = 0.1 95% CI = −0.1–0.9
Handicap	β = 0.3 *p* = 0.07 95% CI = 0–0.5	β = −0.2 *p* = 0.1 95% CI = −0.9–0.1	β = 0.1 *p* = 0.3 95% CI = −0.3–0.8

## Data Availability

The datasets used and analysed during the current study are available from the corresponding author on reasonable request.
